# A pilot longitudinal study of the use of waxy maize heat modified starch in the treatment of adults with glycogen storage disease type I: a randomized double-blind cross-over study

**DOI:** 10.1186/s13023-015-0229-6

**Published:** 2015-02-15

**Authors:** Kaustuv Bhattacharya, Helen Mundy, Maggie F Lilburn, Michael P Champion, David W Morley, François Maillot

**Affiliations:** Charles Dent Metabolic Unit, National Hospital for Neurology and Neurosurgery, WC1N 3BG London, UK; Discipline of Paediatrics and Child Health, The Children’s Hospital at Westmead Clinical School, University of Sydney, Sydney, Australia; Evelina London Children’s Hospital; St Thomas’s Hospital, SE1 7EH London, UK; Department of Epidemiology & Biostatistics, Imperial College, SW7 2AZ London, UK; CHRU de Tours, 37044 Tours, Cedex France

**Keywords:** Glycogen storage disease, Corn-starch, Glycemic index, Carbohydrate, Glycosade, Insulin, Glucose, Lactate, Waxy maize

## Abstract

**Background:**

Uncooked corn-starch (UCCS) has been the mainstay of therapy for the hepatic glycogen storage diseases (GSD) but is not always effective. A new starch (WMHMS) has demonstrated a more favourable short-term metabolic profile.

**Objective:**

To determine efficacy and safety of a new uncooked starch (WMHMS) compared to UCCS over 16 weeks treatment with each.

**Method:**

A double-blind cross-over study of 10 adults (aged 16 – 38 years, six male) with GSD Ia and Ib. After an individualised fast, subjects were randomised to take a 50 g starch-load of either WMHMS or UCCS. Starch-loads terminated when blood glucose was < 3.0 mmol/L or the subject felt subjectively hypoglycaemic. Anonymous biochemical profiles were assessed by 2 investigators and a starch administration schedule recommended. Each starch was delivered in coded sachets and intake was monitored for the following 16 weeks. After a washout period, the protocol was repeated with the alternative product.

**Results:**

4 subjects failed to establish therapy on the cross-over limb. Data from 7 paired starch load showed: longer median fasting duration with WMHMS (7.5 versus 5 hours; p = 0.023), slower decrease in the glucose curve (0.357 versus 0.632 mmol/hr p = 0.028) and less area under insulin curves for the first 4 hours (p = 0.03). Two of six subjects took 50% or less WMHMS compared to UCCS and one took more. Plasma triglycerides, cholesterol and uric acid were unchanged after each study phase.

**Conclusion:**

WMHMS leads to significant reduction in insulin release and reduced starch use in some GSD patients.

## Background

The glycogen storage diseases (GSDs) comprise a group of rare inherited disorders of glycogen metabolism. GSD I (OMIM 232200) is caused by reduced activity of glucose-6-phosphatase (G6Pase, EC 3.1.3.9); GSD Ia by deficiency of the hydrolytic enzyme; and GSD Ib by deficiency of the endoplasmic reticulum transmembrane glucose 6-phosphate transport protein, G6P translocase. The major metabolic consequence of ineffective function of G6Pase is hypoglycaemia, provoked by relatively short fasts. Secondary metabolic disturbances include hyperlactataemia, hyperuricaemia and hyperlipidaemia [1].

Maintaining blood glucose concentration in the normal range improves secondary biochemical features as well as clinical parameters such as growth. The introduction of continuous nasogastric glucose polymer feeds showed this clearly [2,3]. The subsequent introduction of uncooked corn-starch (UCCS) into the daily dietary treatment at least matched this improvement and improved the quality of life of many patients [4,5]. Whilst the introduction of UCCS has benefited many, its use does have problems. For some the duration of normoglycaemia can be less than 4 hours, many find the mixture neither palatable nor convenient, and for others there can be symptoms of bloating, flatulence and diarrhoea with large doses [6]. UCCS is only partially utilized and can be associated with malabsorption in GSD I. [7]. Diarrhoea may also be a feature of GSD I itself or its treatment with corn-starch, and inflammatory bowel disease is a feature of GSD Ib [8].

Apart from sustained normoglycaemia without excessive insulin rise, the features of an ideal starch for treatment of patients with the hepatic GSDs include suppression of secondary biochemical abnormalities, palatability, convenience, few side-effects and maintenance of normal appetite (without excessive weight gain [9,10].

A heat modified waxy maize starch (WMHM20), (Glycologic Ltd, Glasgow, UK), prepared in an emulsified form (WMHMS) (Vitaflo Ltd, Liverpool, UK) has shown improvement in the duration of normoglycaemia and glucose profile in particular of patients with glycogen storage disease type Ia and Ib, [11,12], but there are no data reported on the sustained use of the starch in the normal dietary regimen. This trial investigated the use of this starch (WMHMS) in the daily dietary regimen of adults with glycogen storage disease type Ia and Ib over 16 weeks.

## Methods

The study protocol was approved by the ethics committee nominated by the UK Central Office for Research Ethics Committees (COREC). All adults that attended the Charles Dent Metabolic Unit clinic, London UK with a enzymatic and/or molecular proven diagnosis of GSD Ia or Ib, that were taking uncooked corn-starch as part of their dietary regimen were invited to participate. All recruited subjects gave written informed consent to participate.

The study had a randomised double-blind cross over design. Subjects were allocated an anonymous reference number and were randomly allocated to receive either UCCS or WMHMS. Each starch was manufactured using food-grade techniques and packaged in identical sachets bearing a reference code. The patient reference numbers and sachet reference code were paired by Vitaflo Ltd and the supervising physicians (KB, FM) were blinded to this pairing. The supervising physicians devised a safe personalised fasting period for each patient, prior to each starch load based on previous corn-starch loads and medical history.

### Starch load test

Patients attended in the morning after their individualised fast. An intravenous cannula was placed in the patient’s arm and baseline blood samples were collected. When the participant’s blood glucose was below 4.5 mmol/L, 50 g of the nominated starch mixed in cold water was taken orally. Blood samples were performed at 30 minute intervals for the next 120 minutes and hourly thereafter. No further intake, apart from drinking water, was allowed. The starch load test ended when the patient had fasted for 10 hours, the plasma glucose was ≤3.0 mmol/L on the bedside glucose analyser (YSI 2300 - Yellow Springs, Ohio, USA) or the patient wished to end the test. When the blood glucose was ≤ 3.5 mmol/L, blood tests were performed at 30 minutes intervals until the test end.

### Biochemical data

Plasma was extracted from whole blood samples immediately at the bedside. Plasma glucose and lactate were analysed immediately (YSI 2300 - Yellow Springs, Ohio, USA). Serum to measure insulin was frozen within 30 minutes of collection and subsequently thawed and analysed by a solid-phase, two-site chemiluminescent immunometric assay (Immulite 2000, Diagnostic Products Corporation, Los Angeles, USA). At baseline, “fasting” cholesterol, triglycerides and uric acid were taken.

### Repeat starch load

Data from the first starch load was used to guide management using the particular starch for the subsequent 16 weeks. After a further washout period of 2 – 4 weeks on normal treatment, participants re-attended for a further starch load with the alternative starch.

### Prescription of starch

The glucose and lactate data from the load test were interpreted by two investigators who have experience in interpreting these results (PJL and MPC). Neither of these investigators had any other subject-related role in the study. They were blinded to the identity of both the participant and the starch. They were given some background information of normal starch dosing and the last available corn-starch load data for that participant. On the basis of this information a safe frequency of daytime dosing for the given starch was discussed, agreed and prescribed. The participants were asked to take the nominated starch for 16 weeks. If the volunteer took an overnight infusion of glucose polymer, this was not altered.

### Delivery of starch

The starch was delivered in boxes with individual sachets bearing a reference code identifying the type of starch only to representatives from Vitaflo Ltd. The participants were asked to take the nominated starch as recommended by the blinded prescribers. However, it is recognised that some patients take less or more starch doses in the home environment – the participant was asked to note what they actually took and this was noted by a telephone call made by one member of the research team on a weekly basis.

### Monitoring of starch use and symptoms

For the 16 week study period, participants were asked to keep a record of subjective episodes of hypoglycaemia. Whilst measurement of blood glucose was not recommended, participants were also asked to keep a record of any blood glucose measurement that was taken. One member of the research team contacted the participant to note these data as well as general information about symptoms and appetite over the preceding week. If the participant had an inter-current illness such as diarrhoea or vomiting necessitating additional glucose polymer use or was admitted to hospital for intravenous dextrose, the entire week’s data for this period was omitted from analysis. A maximum of 2 consecutive weeks and 4 total omitted weeks were allowed, from each 16 week period, to remain included in the trial. The participant was asked to complete a diet diary after they had taken the nominated starch for at least 6 weeks. They were asked to write down everything they ate for a 3 day period and the nutritional content of the diet including the starch taken were analysed by an experienced state registered dietician (MFL) using specialised software (Dietplan 6, Forestfield Software, Ltd. Horsham, UK.)

### End of study period

At the end of the 16 week study period, the research volunteer was asked to attend an outpatient clinic. They were weighed and examined and “fasting” plasma triglycerides, cholesterol and uric acid were taken. This was followed by a washout period of 2 – 4 weeks and the patient re-attended for a starch load with the alternative starch. Pre-starch-load preparation including fasting period and assessments were identical. The subject was asked to take the same meals at the same times the day before the second starch-load as they had for the first one.

### Statistics

**Figure 1:**
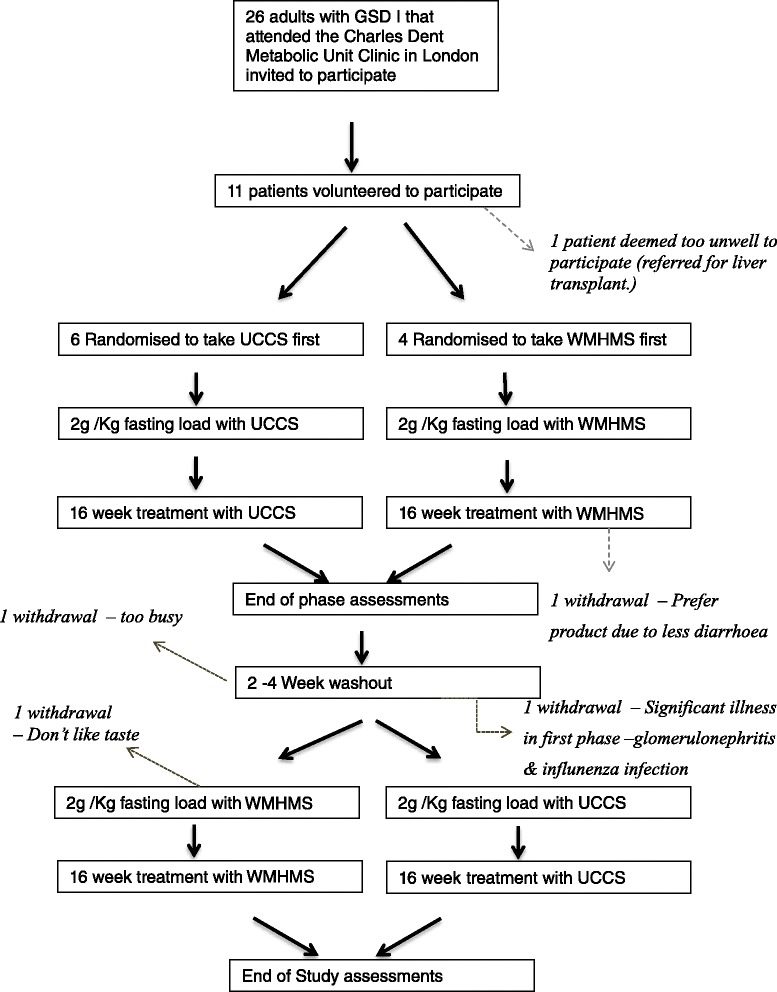
**Indicating trial outline and withdrawals.**

**Figure 2:**
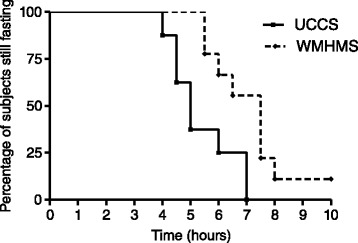
**Kaplan Meier curve indicating test durations for all starch loads performed: n=8 for UCCS and n = 9 for WMHMS.**

**Figure 3:**
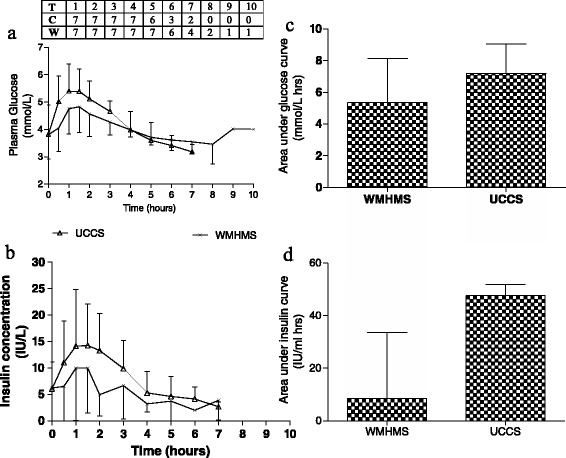
**Mean (+ /- SD) profile for seven paired starch loads for a) glucose and b) insulin.** The median and interquartile range of areas under the curve for the first four hours of the study for **c)** glucose and **d)** insulin. Table for 3a and b indicates number of subjects still fasting at a given time, T in hours, for C = UCCS and W = Waxy maize heat modified strach. (Glucose area calculated above a baseline of 3.0 mmols/L).

Data were entered into GraphPad Prism software (version 5.02, La Jolla, California.) All analyses were two-tailed. Test durations were compared both paired and unpaired by Wilcoxon’s sign rank test and the unpaired data plotted on a Kaplan-Meier curve as shown in Figure 1. The one subject that fasted for 10 hours without evidence of hypoglycaemia was right censored for that starch load. Given the small number of subjects, paired non-parametric analyses (Wilcoxon sign rank test) were used to compare glucose, lactate and insulin profiles. Similarly, areas under the insulin and glucose curve for each individual for the first 4 hours for each starch were compared by paired non-parametric analyses. Areas under the curve were calculated for each patient by GraphPad Prism and checked independently manually by another researcher (DJW). The first four hours of the starch load were used as a cut-off for area calculations for two reasons: a) the mean profile for glucose “cross-over” at this time point with the WMHMS curve were lower than UCCS in the first four hours and higher subsequently (Figure 2) – this has also been noted previously (Bhattacharya et al [11]), and subjects withdrew due to hypoglycaemia after four hours and there were consequently incomplete paired data after this time-point. Weekly quantity of starch intake was compared by a two tailed paired *t*-test for each individual. Total energy intake and energy intake derived from both treatment and dietary carbohydrate were obtained from analyses of dietary diaries and compared by paired non-parametric analyses (Figure 3), as was the difference from baseline of monitoring biochemistry (Table 1). Data were considered statistically significant when p < 0.05.

**Table 1:** **Biochemical monitoring of six patients that completed both phases of trial indicating median baseline value and median change from baseline**

		**Triglycerides (mmol/L)**	**Cholesterol (mmol/L)**	**Uric acid (μmol/L)**	**ALT IU/L**	**CRP mg/L**
	Baseline	3.62	4.7	402	38.6	5
WMHMS	*Median change*	*0.22*	*0.29*	*-41*	*-9*	*0*
	Baseline	5.12	5.35	364	36.8	11
UCCS	*Median change*	*-1.57*	*-0.5*	*-58*	*-13*	*-3*
	Normal Range	0.42 - 2.00	3.35 - 6.2	149 - 369	<50	<10
	**Statistic (p value)**	**0.56**	**0.19**	**0.84**	**0.84**	**0.44**

Paired non parametric analyses of the change from baseline for each individual were performed to produce the statistic indicated in bold. (ALT: Alanine transaminase; CRP: C-reactive protein).

## Results

10 volunteers were recruited to the study. Patient details are shown in Table 2 and the disposition of recruited patients indicated in Figure 1. A total of 4 patients withdrew from the trial protocol of which 3 did not have a repeat starch load. Paired data were therefore available for 6 subjects taking each product for 16 week phase of the trial and for 7 starch loads.

**Table 2:** **Demographics of patients, indicating normal daily therapy and whether they were successfully blinded in this study**

**ID**	**Age (yrs)**	**Sex**	**GSD type**	**BMI**	**Normal starch treatment**	**Medications**	**Complications**	**Blinding success**
A	23	M	1a	21.8	D – 120 g UCCS N –125 g polymer	Allopurinol,Ramipril, Carbamazepine Multivitamins	Seizures (stable)	Yes
B	24	F	1a	26.0	D – 60 g UCCS N – 55 g UCCS	Allopurinol, Ramipril. Vit B12	PCOD Renal impairment	No – able to tell difference of taste.
C	25	F	1a	26.7	D – UCCS 160 g N - 120 g Polymer	Allopurinol Multivitamins	PCOD	Yes
D	38	M	1a	22.3	D –UCCS 200 g N – UCCS 100 g	Allopurinol simvastatin, codeine, lansoprazole	Hepatic adenoma (removed) Chronic scar pain Heartburn	No – took much less WMHMS than UCCS
E	16	M	1b	27.1	D – 160 g N – 175 g polymer	Allopurinol	None	No – Did not like taste of WMHMS to start but habituated
F	17	M	1b	34.6	D – 120 g N – 140 g polymer	Allopurinol	Obese	Yes – Thought UCCS was test starch.
G	19	F	1b	25	D – 90 g N – 120 g Polymer	Allopurinol GCSF	Anaemia Recurrent infections	No – able to detect different texture
H	18	F	1b	24.9	D – 180 g N – 120 g	Allopurinol, GCSF Co-trimoxazole Thalidomide	GSD IB enterocolitis Bronchiectasis	No – withdrew as did not like taste of WMHMS
I	24	M	1b	19.7	D – 160 g N – 80 g	Allopurinol GCSF	Short stature Diarrhoea	No – took much less WMHMS than UCCS.
J	36	M	Ib	22.2	D- 30 g N- 30 g	Allopurinol Ferrous sulphate	Hepatic adenoma Chronic anaemia	No – had fewer episodes of diarrhoea with WMHMS and refused to take UCCS

D = day; N = Night; BMI – Body mass index; GCSF – granulocyte colony stimulating factor; PCOD – poly-cystic ovarian disease.

### Adverse events

Whilst on UCCS, 4 of 8 subjects were admitted into hospital – all for reasons not deemed to be trial related – one for dysphagia related to a substance ingested by mistake, a second one for haematuria and abdominal pain probably related to a renal stone (this subject withdraw from the second limb of the study on WMHMS) and the remaining two for vomiting associated with hypoglycaemia during an inter-current viral illness. Whilst on WMHMS, 1 subject with GSD Ib was admitted twice to hospital, firstly with post streptococcal glomerulonephritis and secondly with a viral lower respiratory tract infection (this subject subsequently withdrew without taking UCCS), and the complications not deemed to be trial related.

### Starch loads

8 starch loads were performed using UCCS with a median duration of 5 hours and 9 starch loads with WMHMS with a median duration of 7.5 hours (p = 0.0091, ratio 0.67, CI of ratio 0.29,1.04. – Figure 2). When only the 7 paired starch loads are considered, the median durations remain the same and the comparison were still statistically significant, (p = 0.023). The mean glucose and insulin profiles are indicated in Figure 3. The insulin curve for WMHMS was below the UCCS curve for most of the 7 hours that serum insulin was measured (p = 0.014), reflecting the fact that the glucose curve for WMHMS was also below UCCS for the first 4 hours of the study (p = 0.016) and then was higher than the UCCS curve. There was a trend for the area under the WMHMS glucose curve to be lower; the median area and inter-quartile range of the area above a baseline of 3.0 mmol/L for WMHMS being 5.36 mmol/L hrs (3.56 – 8.12,) and for UCCS being 7.20 mmol/L hrs (6.01 – 9.86) (p = 0.10) for the first four hours of the study. Similarly the area under the insulin curves was significantly lower for WMHMS, being 114 pmol/L hrs (70.1 – 289) compared to UCCS being 386 pmol/L hrs (123 – 415) (p = 0.031), as indicated in Figure 3. Similar to previous studies, there was no difference between either the lactate profile or the area under the lactate profile for the first four hours: WMHMS being 21.9 mmol/L hrs (8.25 – 34.6) and UCCS 18.3 mmol/L hrs (16.8 – 27.2), (p = 0.47).

### Use of starch during trial

**Figure 4:**
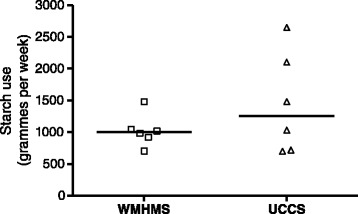
**Mean weekly trial starch use over 16 weeks for 6 patients with paired data.** Median of these indicated by bold line.

Mean weekly starch use by the 6 patients that completed the study is shown in Figure 4. Whilst the differences for the group are not statistically significant, 2 subjects did take significantly less WMHMS than UCCS when their weekly intakes were compared by paired *t*-tests (920 g vs 2600 g), and (980 vs 2100 g), p < 0.001 for both. One subject took more WMHMS than UCCS (1020 g vs 690 g p < 0.001), but this was related to better compliance with prescribed therapy after she was notified in the washout period about deteriorating long-term renal function. This was substantiated by an increase of 4.3 Kg weight over the washout period on normal therapy prior to starting WMHMS. The reduction of starch use in two subjects whilst taking WMHMS was not matched by broadening of dietary intake. As a consequence their total energy intake reduced from 2410 and 3044 Kcal/day to 1876 and 2454 respectively. There was no difference in median weight change between the 2 populations: + 0.25 Kg for WMHMS and – 0.05 Kg for UCCS. Patient specific data is indicated in Table 3. Those that took less WMHMS lost weight. Two patients gained over 6 Kg of weight taking WMHMS; patient B was more compliant with diet after receiving adverse GFR tests results in the wash-out period and consequently took approximately 150% of the amount of starch in the second phase of the study on WMHMS. Patient E found WMHMS unpalatable adding chocolate powder to this and not to UCCS leading to weight gain on the WMHMS phase. Biochemical monitoring was also satisfactory as shown in Table 1. Symptoms that were monitored on a weekly basis were general well-being, appetite, abdominal bloating, diarrhoea, symptomatic hypoglycaemia and measured hypoglycaemia. For the whole group, there was no difference in all parameters but there were some individual variations. One patient was more bloated with UCCS, but this subject took more UCCS than WMHMS, and the bloated sensation may have reflected increased total starch intake. There was no difference with other patients. Several patients reported marginally improved appetites on WMHMS, but this was not substantiated by objective assessments of dietary records. Of the 9 subjects that had taken WMHMS in this study, 7 opted to continue therapy after the trial concluded. Table 2 indicates whether the blinding was successful and in some cases it wasn’t. Several of the patients commented on the texture and mouth-feel of WMHMS being different to UCCS with a more gritty texture to that which they were habituated to. Some patients recognized and overcame this texture difference whilst others didn’t and withdrew.

**Table 3:** **Weight in Kg before and after the 16 week phase of each starch treatment with change of weight indicated over the period indicated**

**ID**	**First**	**Pre**	**Post**	**Post**	**UCCS**	**Pre**	**Post**	**Post**	**WMHMS**
	**Starch**	**UCCS**	**UCCS**	**- pre**	**Use g/week**	**WMHMS**	**WMHMS**	**- pre**	**Use g/week**
A	W	57.8	58	*0.2*	**1033**	56.7	57.7	*1*	**1047**
B	U	63.2	60.8	*-2.4*	**688**	65.1	72.2	*7.1*	**1018**
C	U	77.6	76.4	*-1.2*	**1480**	76.5	76	*-0.5*	**1478**
D	U	55	59.6	*4.6*	**2648**	59.5	55	*-4.5*	**920**
E	U	73.1	72.8	*-0.3*	**702**	73.6	80.5	*6.9*	**703**
I	W	50.3	52.3	*2*	**2100**	53.2	50.4	*-2.8*	**983**

The amount of treatment starch taken in grammes per week is also shown in bold. See text for explanations of individual differences. The starch to which the patient was first randomized to is indicated in the second column. W = WHMHMS and U = UCCS.

## Discussion

The dietary regimen for the treatment of type I glycogen storage disease has previously been called the “intensive regimen”, with hypoglycaemia a predominant feature after even quite short fasting intervals [13,14]. For some, the intensive regimen has improved after the introduction of uncooked corn-starch, but for others fasting tolerance can still be as little as 4 hours. As a consequence several need disruptive overnight doses of starch. Several patients can have gastrointestinal disturbances including bloating flatulence and diarrhoea [15,16]; colonic fermentation of unutilised corn-starch could be partly responsible for this in both GSD Ia and Ib, although inflammatory bowel disease is a well-recognised feature of GSD Ib [8].

### Glycemic Load of WMHMS

Waxy maize starch differs from typical corn-starch in that it has a high proportion of amylopectin. It has been subject to research interest due to its low glycaemic index [17]. Chemical or physical modification appears to alter the physiological responses further [18,19]. Using this type of approach, our research group was able to demonstrate a longer median duration of normoglycaemia for patients with GSD with a heat modified waxy maize starch compared to uncooked corn-starch [11]. However, this study was confounded by several factors including the age, type of GSD and precision of bedside glucose measurements. As a consequence the most meaningful result was that the glucose decreased slower with the new starch compared to traditional uncooked corn-starch. These findings were sufficient to trigger another independent study, which has replicated these data [12]. Methodologically the latter study tested a more homogeneous group of patients at rest overnight, thereby limiting energy expenditure. Both studies aimed to use large doses to gauge whether a single night time dose would suffice for metabolic homeostasis. The current study differed in that smaller doses were used (50 g in adults– 41 g of carbohydrate), that could be incorporated into the standard daytime dietary regimen. Compared to our previous study, a more homogeneous group of patients were selected in that all were adults with GSD Ia or Ib and reliable glucose and lactate measurements were available at the bed-side. The combination of these and other factors have resulted in this study being better able to define the glucose and insulin responses to each starch load. Of note, the studies of Jenkins et al that defined glycaemic index indicated that greater relative differences in glycaemic index were observed when 50 g of a carbohydrate was used compared to 100 g [20]. These studies used frequent glucose measurements over 120 minutes with subjects fasting in a standardised way before the carbohydrate was administered. We also attempted to control baseline by administering the starch when the plasma glucose was below 4.5 mmols/L and sampled at 30 minute intervals for the first 120 minutes. There was consequently better resolution of the glycaemic response compared to the other studies of this starch in GSD. The significantly reduced area under the insulin curve using WMHMS is consistent with the lower glucose curves in the first four hours, with subsequent slower decline in glucose. As mentioned by Correia et al, this is a safer profile with a lesser tendency to sudden hypoglycaemia. The aim of researchers that manage GSD is to extend the period of normoglycaemia rather than decrease the early insulin response to carbohydrate ingestion, but it is clear for this starch that the latter affects the former. The duration of normoglycaemia was longer for the new starch compared to UCCS replicating the findings of the studies using higher doses. However, this finding should not be over-interpreted as some individuals wished to end the test before documented hypoglycaemia was identified. As in previous studies with GSD, this probably occurs because patients are unfamiliar with fasting for 7 hours or greater and could consequently feel insecure. However, if replicated, this should translate in to fewer starch doses taken by individuals and less overall starch use.

### Methodological difficulties

Only two individuals took significantly less WMHMS than UCCS over the course of this study. Part of the reason for this was because of the safety aspect of the study design. Three individuals that took overnight pump feeds of glucose did not have this aspect of their treatment altered. Consequently, there was a reduced period of 14 or 15 hours in which any dose of studied carbohydrate could be taken. The only real options in that time period were to prescribe either two doses at 7 hour intervals or three doses at 4 – 5 hour intervals; for each of these patients, the more conservative 4 hourly regimen was selected for both starches, even if the data suggested someone could fast for six hours comfortably. These subjects, who place naso-gastric tubes themselves every night, were particularly compliant, only ever taking more than the prescribed dose, but never less. Therefore, no difference is seen for these 3 individuals. By contrast, the 3 subjects who take corn-starch both day and night had more flexibility with prescriptions and also varied the amount they took more. Two of these three subsequently took significantly less WMHMS and the other subject complied more with the prescribed dose due to adverse long-term deterioration in renal function.

### Further studies

The mechanism of potential extended duration of WMHMS needs more investigations. Our previous study [11], demonstrated increased fermentation of UCCS, confirming previous findings, but this was not matched by increased enrichment of ^13^CO_2_ with WMHMS. This may have been confounded by the age range of subjects and the variable duration of tests. Sustained incretin release after UCCS ingestion has been shown when compared to other carbohydrates. This may also account for increased insulin release when UCCS is compared to waxy maize starch [21]. There are also natural enrichment data demonstrating delayed gastric emptying of waxy maize starch when compared to other carbohydrates [17]. It is probable, but not definite, that the heat modified waxy maize starch behaves more like its native starch than UCCS. As such, delayed gastric emptying and diminished insulin release are potential mechanisms of action. The role of appetite suppression modulated by adiponectin and the ghrelin and leptin axis has not been sufficiently studied in GSD. It is possible, in these fasting GSD studies, that patients mistake hunger for hypoglycaemia because patients are unfamiliar with prolonged fasting and its associated physiology.

### WMHMS in healthy volunteers

WMHMS has now been studied in healthy volunteers allowing its role to be investigated under different physiological stresses to patients with GSD. Roberts et al studied 9 male competitive cyclists during and after intense exercise [22]. They compared WMHMS with maltodextrin finding decreased insulin production and increased fat oxidation in the former group. Bracken et al compared WMHMS (Glycosade ^TM^) with UCCS (Argo ^TM^) and dextrose at rest, followed by an hour of exercise and a subsequent 2 hour recovery period [23]. In the resting phase, peak glucose concentration occurred later with WMHMS compared to UCCS and there was evidence of increased lipid oxidation during exercise too. Conversely, there was equivalent increased carbohydrate oxidation in exercise of UCCS and dextrose compared to WMHMS. It is very difficult to extrapolate these data to the GSD population. Firstly it is important to discriminate whether glucose oxidation is from endogenous or exogenous sources – if the increased carbohydrate oxidation is endogenous, this would be impaired in GSD and lead to compromise. Conversely, some individuals with GSD appear to have impairment in ketone body production so increased lipid oxidation may not be replicated in GSD patients [24].

### Context of these data in patients

The significant lifelong carbohydrate intake that these patients take put them at particular risk of insulin resistance and non-insulin dependent diabetes mellitus, although there are few reports of these complications [25]. Data from our dietary surveys demonstrate that some patients take more carbohydrate than recommended for the population and theoretically required in GSD patients [24]. There is also a tendency of some individuals to take higher glycaemic index commercially available drinks to prevent or treat hypoglycaemia. This results in additional fluctuation of glucose and insulin over and above that caused by prescribed dietary products. Complications such as hyperlipidaemia, hepatic steatosis and polycystic ovarian disease are well recognised features of the hepatic glycogenoses and it is likely that insulin resistance plays some part in the pathophysiology of such [6,26-28]. Long-term reduction in insulin release by using a starch such as WMHMS may therefore result in health benefits, although data need to be prospectively gathered over many years to substantiate this. Quite apart from having benefits for GSD-I patients, WMHMS may be of benefit in other disorders that require sustained euglycaemia without excessive insulin release. The dietary treatment of diabetes mellitus could well benefit from this therapy, but again this needs to be tested independently. Other inborn errors of metabolism associated with hypoglycaemia may also benefit from this treatment. The role of WMHMS in fat oxidation defects needs to be cautiously evaluated as insulin release may be protective in these disorders.

## Conclusion

This study with a small number of patients with GSD Ia and Ib, demonstrated that WMHMS can be safely incorporated into the dietary regimen of these patients. Our experience shows that these patients are a difficult group to study. From a metabolic perspective, they are brittle with a tendency for hypoglycaemia after very short fasts with marked fluctuation of related variables, insulin and lactate, as well as longitudinal indices of metabolic control such as triglycerides and uric acid. These study difficulties have been commented on specifically on a meta-analysis of GSD Ia dietary therapy [29]. Co-morbidities such as diarrhoea, renal stones, renal impairment, inflammatory bowel disease and recurrent infections need to be taken into account for long-term studies and hospitalisations are not infrequent as was observed in this trial period. There may well be an ascertainment bias as the sickest patients are more likely to want to change their life, whereas several of the stable patients were not keen to participate. Most previously published studies of dietary interventions in this disorder have, therefore examined short-term or retrospective data, rather than using a randomised controlled trial design [4,14,30,31]. Any future trial should recognise these problems in their design. The dose selected in this study was one that is commonly used by patients treated with corn-starch but the optimal dose of WMHMS may well be different. As a consequence, formal dosing studies should be performed to ascertain the best treatment regimen of the new starch. It was our hope that any reduction in starch use would be matched by a broadening of dietary range approaching recommendations for peers. This aspect of the study was observational and no such alterations took place, with the 2 subjects taking less starch, taking less overall energy. This may reflect the narrow dietary range that these subjects have learnt to adhere to, as well as the relatively short length of the study. Dietary advice has been given after the conclusion of this trial, in the hope that a broader range of foods would be taken. Typically many patients have a set dietary routine and repertoire of responses to hypoglycaemia from which they find it difficult to deviate [24,32]. As a consequence, dietary advice in GSD I should be dynamic and supportive, incorporating physiological changes that occur over time but still understanding patients’ resistance to change.

This study has demonstrated an improved glucose and insulin profile after 50 g of WMHMS was given when compared to UCCS. The study provides preliminary data that the starch can be safely introduced into the dietary regimen of patients without untoward effects. Further studies determining mechanism of action, role in other disorders, optimal dosing and long-term effects should be performed.
